# Imaging‐Based Prediction of Ki‐67 Expression in Hepatocellular Carcinoma: A Retrospective Study

**DOI:** 10.1002/cam4.70562

**Published:** 2025-02-18

**Authors:** Chiyu Cai, Liancai Wang, Lianyuan Tao, Hengli Zhu, Yongnian Ren, Deyu Li, Dongxiao Li

**Affiliations:** ^1^ Department of Hepatobiliary and Pancreatic Surgery Zhengzhou University People's Hospital Zhengzhou China; ^2^ Department of Digestive Diseases Zhengzhou University People's Hospital Zhengzhou China

**Keywords:** machine learning, nomogram, prognosis, radiomics, risk scorecard, survival

## Abstract

**Aim:**

This study aims to develop a non‐invasive, preoperative predictive model for Ki‐67 expression in HCC patients using enhanced computed tomography (CT) and clinical indicators to improve patient outcomes.

**Methods:**

This retrospective study analyzed 595 post‐curative hepatectomy HCC patients. Patients were categorized into high (> 20%) and low (≤ 20%) Ki‐67 expression groups based on cellular proliferation levels. Radiomic features were extracted from enhanced CT scans and combined with clinical parameters to develop a predictive model for Ki‐67 expression.

**Results:**

Key clinical factors impacting Ki‐67 expression in HCC included alpha‐fetoprotein (AFP), non‐smooth tumor margin, ill‐defined pseudo‐capsule, and peritumoral star node. From 1441 initially extracted radiomic features, 16 key features were selected using Lasso regression. These features were used to develop a radiomics model, which, when combined with clinical data, yielded an integrated predictive model with high accuracy. The combined model achieved an area under the curve (AUC) of 0.854 in the training group and 0.839 in the validation group. A nomogram based on this model was constructed, and its predictive accuracy was validated through calibration curves and decision curve analysis. A risk scorecard model was also constructed as a practical tool for clinicians to assess the risk level of high Ki‐67 expression, facilitating personalized treatment planning. Survival analysis demonstrated significant differences in 3‐year overall survival (OS) and progression‐free survival (PFS) rates between patients with high and low Ki‐67 expression, indicating the model's strong prognostic capability.

**Conclusions:**

This study successfully developed a comprehensive model that integrates radiomic and clinical data for the preoperative prediction of Ki‐67 expression in HCC patients.

## Graphical abstract

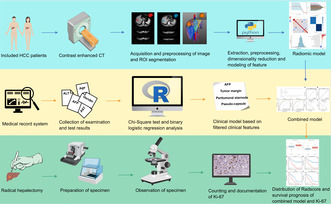

**Abbreviations**
AFP: alpha‐fetoprotein
AI: artificial intelligence
ALB: albumin
ALT: alanine aminotransferase
AST: aspartate aminotransferase
AUC: area under the curve
CEA: carcinoembryonic antigen
CI: confidence interval
CT: computed tomography
ES grade: Edmondson‐Steiner grade
GLCM: gray level co‐occurrence matrix
GLDM: gray level dependence matrix
GLRLM: gray level run length matrix
GLSZM: gray level size zone matrix
HCC: hepatocellular carcinoma
ICC: interclass correlation coefficient
MRI: magnetic resonance imaging
NGTDM: neighboring gray tone difference matrix
NLR: neutrophil‐to‐lymphocyte ratio
OR: odds ratio
OS: overall survival
PFS: progression‐free survival
PLR: platelet‐to‐lymphocyte ratio
PLT: platelet count
PT: prothrombin time
RBC: red blood cell count
ROC: receiver operating characteristic
SVM: support vector machine
TBil: total bilirubin

## 1 Introduction

Liver cancer, particularly hepatocellular carcinoma (HCC), poses a significant global health challenge, ranking as the sixth most common cancer globally and the third leading cause of cancer‐related death [1]. Despite advancements in treatments, including surgery, interventional therapies, targeted therapies, and immunotherapies, HCC's high recurrence and metastasis rates continue to impair patient survival and quality of life [2, 3]. Ki‐67 protein, a critical biomarker of cellular proliferation, is significantly overexpressed in several cancers [4, 5, 6], including HCC. In HCC, elevated levels of Ki‐67 not only signify increased proliferative activity but also serve as an independent prognostic factor essential for evaluating patient outcomes [7, 8]. Thus, accurate evaluation of Ki‐67 expression is essential for prognosis and treatment planning.

However, current methods for detecting Ki‐67 in HCC, such as post‐surgical pathology or pre‐surgical biopsy, are invasive, pose a risk of complications, and often rely on limited tissue samples, which may not fully capture the tumor's heterogeneity. These limitations can hinder comprehensive preoperative assessment and delay the formulation of optimal treatment strategies. A non‐invasive prediction of Ki‐67 expression would reduce the need for invasive procedures and provide a more comprehensive understanding of the tumor's proliferative activity, thereby supporting individualized treatment strategies.

Recent advancements in medical imaging and artificial intelligence (AI) have catalyzed extensive research in this field [9, 10, 11]. These studies focus on applying deep learning and traditional radiomics to extract features from magnetic resonance imaging (MRI) and computed tomography (CT) images, aiming to predict Ki‐67 expression in HCC [12, 13, 14, 15]. However, the reliance on small sample sizes in existing literature restricts the generalizability and accuracy of these findings. In medical imaging research, particularly for heterogeneous diseases like HCC [16, 17], large‐scale studies are essential to increase statistical power and enhance the reliability and applicability of results.

This study aims to leverage a larger cohort to evaluate the effectiveness of enhanced CT and clinical indicators in predicting Ki‐67 expression in HCC. By integrating imaging and clinical data, we seek to develop a predictive model that is more accurate and reliable than current biopsy‐based approaches. Such a model would provide a more comprehensive view of tumor biology, aid in identifying high‐risk patients, and serve as a valuable tool for preoperative risk assessment and personalized treatment planning.

## 2 Material and Methods

### 2.1 Study Participants

This study was conducted in strict adherence to the ethical guidelines of the Declaration of Helsinki and received approval from the Ethics Committee of Zhengzhou University People's Hospital (Approval No. (2023) Ethical Review 12). As this was a retrospective analysis, the ethics committee granted an exemption from the requirements for informed consent, recognizing that the study involved minimal risk to patients. All patients' data extracted from medical records were handled with strict confidentiality and data protection measures in place. A total of 595 HCC patients who underwent curative hepatectomy between 2018 and 2023 were included. Inclusion criteria were: (1) ages between 18 and 80 years; (2) curative hepatectomy with a pathological diagnosis of HCC; (3) postoperative Ki‐67 testing. Exclusion criteria included: (1) Incomplete clinical and imaging data (*n* = 138); (2) Absence of curative liver cancer resection (*n* = 126); (3) History of other malignancies (*n* = 89); (4) Preoperative antitumor therapy (*n* = 62); (5) Poor image quality (*n* = 24, evaluation criteria are shown in Supporting Information—Data S1). Patients were divided into a training set (*n* = 416) and a validation set (*n* = 179) in a 7:3 ratio through simple random sampling, as illustrated in Figure S1.

### 2.2 Data Collection

Clinical data of the patients included gender, age, levels of alanine aminotransferase (ALT), aspartate aminotransferase (AST), albumin (ALB), total bilirubin (TBil), neutrophil‐to‐lymphocyte ratio (NLR), platelet‐to‐lymphocyte ratio (PLR), red blood cell (RBC) count, platelet (PLT) count, prothrombin time (PT), carcinoembryonic antigen (CEA), alpha‐fetoprotein (AFP), hepatitis B status, and liver cirrhosis presence. Pathological information encompassed the Edmondson‐Steiner grade and the Ki‐67 index. Traditional imaging characteristics included tumor characteristics such as number, diameter, margin, growth pattern, hemorrhage, necrosis, pseudo‐capsule, and peritumoral star node. The evaluation of imaging data was independently conducted by two hepatobiliary surgeons, blinded to the patients' pathological information. Survival data of the patients, including 1‐, 2‐, 3‐year overall survival (OS) and 1‐, 2‐, 3‐year progression‐free survival (PFS), were collected through outpatient visits or telephone follow‐ups until September 31, 2023.

OS is measured from the date of surgery to either the date of the patient's death or the last confirmed follow‐up, whichever comes first. PFS is the duration from the clinical confirmation of complete remission post‐treatment to the first observation of lesion recurrence.

### 2.3 Ki‐67 Index Measurement

The Ki‐67 index was obtained directly from the medical records of Zhengzhou University People's Hospital, evaluated by pathology experts. The process involved fixing HCC surgical specimens in 10% formalin, paraffin embedding, and preparing 4‐μm thick tissue sections for Ki‐67 immunohistochemical staining. The Ki‐67 index was calculated as the proportion of positive cells (brown‐stained nuclei) to the total cell count. HCC patients were categorized into high (> 20%) and low (≤ 20%) Ki‐67 groups, following the literature [18, 19].

### 2.4 Image Data Processing

**FIGURE 1:**
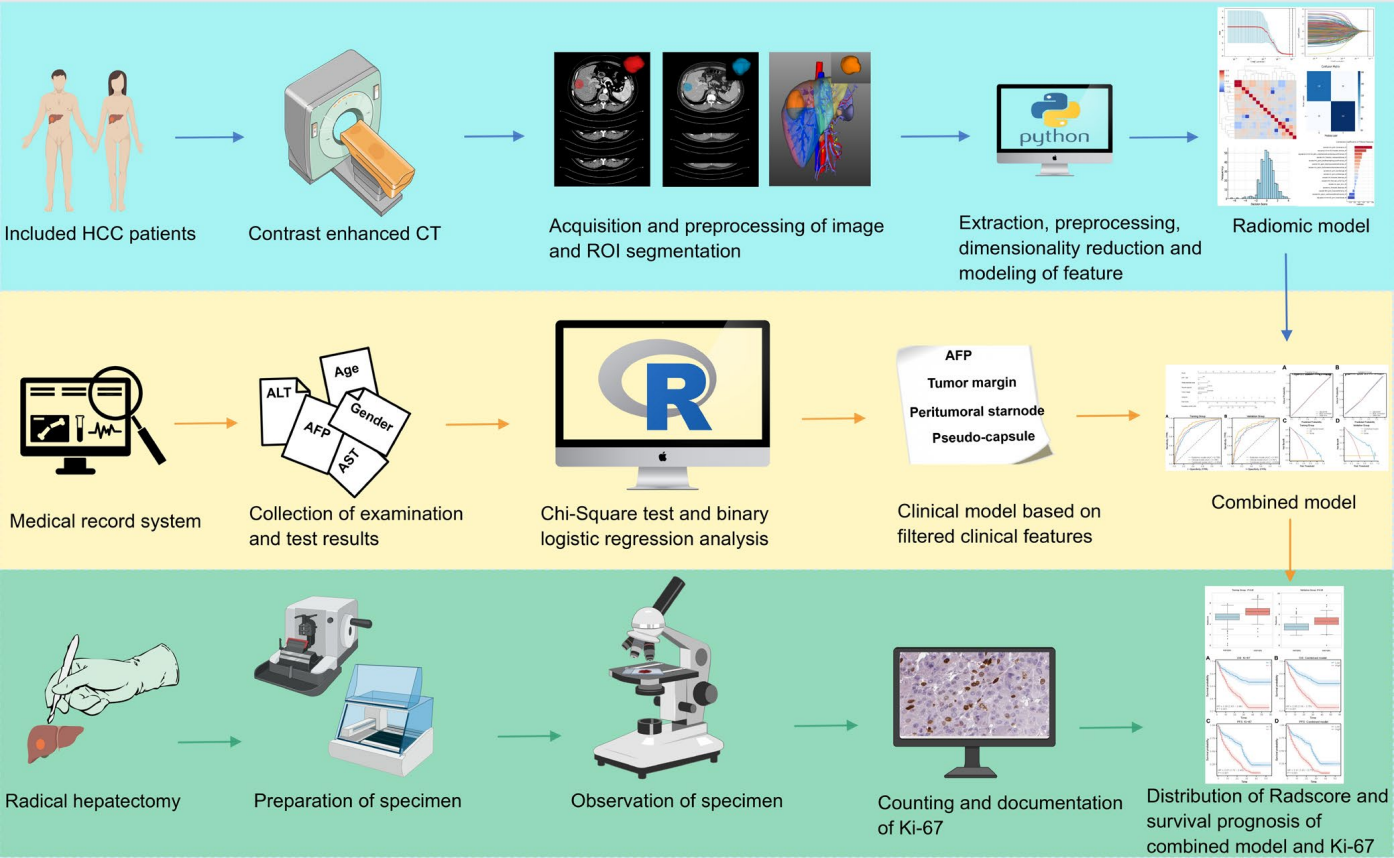
Comprehensive research workflow. Imaging data is acquired through radiomics, including image preprocessing, ROI segmentation, feature extraction, and dimensionality reduction. Clinical data is collected from clinical systems for analysis. Pathological specimens are used for Ki‐67 detection. These multimodal data sources are integrated for predictive model construction, followed by validation and application in survival analysis.

The overall workflow is depicted in Figure 1. All patients underwent abdominal enhanced CT scans; specific equipment and parameters are detailed in the Supporting Information—Data S1. Imaging data in DICOM format were anonymized using DicomCleaner software.

(http://www.dclunie.com/pixelmed/software/macexe/DicomCleanerMac.zip). To minimize the impact of varying CT scanner parameter settings, *Z*‐score normalization was applied to all images using Python's SimpleITK library. This process involved subtracting each pixel value from the mean intensity of the entire image and then dividing by the standard deviation, ensuring a uniform intensity baseline across different images. Furthermore, SimpleITK was also used for standardization and resampling of the images, employing trilinear convolution interpolation to uniformly adjust the voxel size to (1 mm × 1 mm × 1 mm). This step ensured consistent physical dimensions of the voxels across all images, facilitating subsequent feature extraction and analysis. Rigid registration of arterial and venous phase CT images were performed using ITK software (https://itk.org/). Rigid transformations, including rotations around three principal axes and translations along these axes, were applied. A gradient descent optimizer with a learning rate of 1.0 and a maximum iteration limit of 500 was used. Mutual information was utilized as the similarity metric for registration, ensuring maximal alignment between different images. Linear interpolation was employed as the interpolation method to maintain image quality. Initial rotation and translation parameters were automatically calculated based on the image centers. The convergence criterion for the registration process was set such that changes in rotation and translation parameters were below a predefined threshold. The region of interest (ROI) delineation was manually performed in ITK software by a hepatobiliary surgeon with 5 years of clinical and image reading experience, following the guideline based on the clearly visible tumor boundaries on CT images. To assess the consistency of the delineation, another surgeon with 10 years of image reading experience repeated the delineation on images of 50 patients. The interclass correlation coefficient (ICC) was then calculated for the delineations, and only features with an ICC greater than 0.75 were selected for the final analysis.

### 2.5 Feature Extraction and Data Analysis

In the process of radiomic feature extraction, we employed the pyradiomics package (https://github.com/Radiomics/pyradiomics) [20]. The scope of feature extraction was extensive, encompassing original image shape features such as maximum 3D diameter and sphericity, as well as first‐order statistical features that included various measures of pixel intensity. Textural features were predominantly derived from methodologies like the gray level co‐occurrence matrix (GLCM), gray level run length matrix (GLRLM), and gray level size zone matrix (GLSZM). Moreover, wavelet transformations were applied to extract multi‐scale textural features, with the quantization of gray levels set to 25 bins. This approach was designed to enhance the robustness and interpretability of the extracted features, catering to the intricate nature of our dataset. After validating for ICC, eliminating invalid features, handling missing values, and standardizing using the *Z*‐score, 1151 effective radiomic features were selected. This selection includes 273 first‐order statistical features, 17 shape‐based features, 216 features from the GLCM, 216 features from the GLRLM, 192 features from the GLSZM, 45 features from the neighboring gray tone difference matrix (NGTDM), and 192 features from the gray level dependence matrix (GLDM). Dimension reduction in the training set was conducted using Lasso regression (max iterations 5 × 10^6^, regularization parameter 0.04) and 5‐fold cross‐validation. Radscore, used to establish the imaging model, was calculated using a support vector machine(SVM) with a linear kernel. Details of the radiomics model construction using Radscore are provided in the Supporting Information—Data S1. Clinical factors and traditional imaging characteristics were analyzed using univariate and multivariate analyses in SPSS, with statistically significant indicators incorporated into a clinical model for predicting Ki‐67 index.

### 2.6 Combined Model Construction and Validation

A comprehensive model combining clinical and imaging models was constructed. A nomogram model was established in R, with its predictive efficacy validated through calibration curves and decision curve analysis. The model's predictive performance was also evaluated using receiver operating characteristic (ROC) curves, sensitivity, and specificity.

### 2.7 Construction of Risk Factor Scorecard Model

Each risk factor was categorized and assigned a reference value for each category. The score for each group was calculated using the formula provided in the Supporting Information—Data S1. We then used the Youden index to determine the optimal threshold, calculated the linear predictor, and assessed the contribution of each score to the total score based on the model's coefficients and the scorecard values. This process converted the linear predictor to a threshold score on the scorecard. Finally, we developed a convenient method to evaluate the risk score of high Ki‐67 expression.

### 2.8 Statistical Analysis

Data analysis was performed using Python (version 3.12.0), SPSS (version 26.0), and R (version 4.3.2). Categorical variables were presented as counts and percentages, and continuous variables as mean ± SD. The Chi‐square test or Fisher's exact test was used for categorical variables, and the Mann–Whitney *U* test for continuous variables. OS and PFS were estimated using the Kaplan–Meier method. A two‐sided *p*‐value of less than 0.05 was considered statistically significant, with statistical tests chosen based on data distribution and analysis objectives to ensure appropriate and accurate statistical inferences.

## 3 Results

### 3.1 Clinical Characteristics

This study included a total of 595 patients, with 485 males (81.5%) and 110 females (18.5%), and an average age of 58.2 ± 10.3 years. Of these, 317 patients (53.28%) were in the high Ki‐67 group, while 278 (46.72%) were in the low Ki‐67 group. The cohort was divided into a training set of 416 patients and a validation set of 179. In the training set, there were 220 high Ki‐67 patients (52.88%) and 196 low Ki‐67 patients (47.12%). In the validation set, 97 were high Ki‐67 (54.19%) and 82 were low Ki‐67 (45.81%). A comparative analysis of general clinical and traditional imaging characteristics between the two groups revealed no significant differences in Tables S1 and S2 (*p* > 0.05). Univariate and multivariate analyses were performed on the training group, as shown in Table 1, identifying AFP (OR = 1.94, 95% CI:1.16–3.25), tumor margin (OR = 3.04, 95%: 1.93–4.80), pseudo‐capsule (OR = 3.47, 95% CI: 2.22–5.42) and peritumoral star node (OR = 2.92, 95% CI: 1.59–5.38) as significant factors influencing Ki‐67 expression in Figure S2 (*p* < 0.05). Based on these clinical and radiological features, a clinical prediction model was established.

**TABLE 1:** Univariate and multivariate analyses of predictors for Ki67 expression in the training group.

Variable	Training group					
	Univariate analysis			Multivariate analysis		
	Ki‐67 ≤ 20 (*n* = 196)	Ki‐67 > 20 (*n* = 220)	*p*	*β*	OR (95% CI)	*p*
Sex,*n* (%)						
Male	159 (81.1)	176 (80.0)	0.773			
Female	37 (18.9)	44 (20.0)				
Age (years)						
≤ 65	136 (69.4)	170 (77.3)	0.069			
> 65	60 (30.6)	50 (22.7)				
ALT (U/L)						
≤ 40	126 (64.3)	140 (63.6)	0.890			
> 40	70 (35.7)	80 (36.4)				
AST (U/L)						
≤ 40	113 (57.7)	134 (60.9)	0.500			
> 40	83 (42.3)	86 (39.1)				
ALB (g/L)						
≤ 40	98 (50.0)	126 (57.3)	0.137			
> 40	98 (50.0)	94 (42.7)				
TBil (umol/L)						
≤ 17.1	128 (65.3)	143 (65.0)	0.948			
> 17.1	68 (34.7)	77 (35.0)				
NLR, *n* (%)						
≤ 2.5	94 (48.0)	106 (48.2)	0.964			
> 2.5	102 (52.0)	114 (51.8)				
PLR, *n* (%)						
≤ 111.5	100 (51.0)	112 (50.9)	0.982			
> 111.5	96 (49.0)	108 (49.1)				
RBC (×10^12^/L)						
≤ 3	5 (2.6)	7 (3.2)	0.701			
> 3	191 (97.4)	213 (96.8)				
PLT (×10^9^/L)						
≤ 100	45 (23.0)	55 (25.0)	0.627			
> 100	151 (73.0)	165 (75.0)				
PT (s)						
≤ 13	121 (61.7)	139 (63.2)	0.761			
> 13	75 (38.3)	81 (36.8)				
CEA (ng/mL)						
≤ 5	176 (89.8)	195 (88.6)	0.704			
> 5	20 (10.2)	25 (11.4)				
AFP (ng/mL)						
≤ 400	159 (81.1)	135 (61.4)	0.000	0.66	1.94 (1.16–3.25)	0.012
> 400	37 (18.9)	85 (38.6)				
HBsAg						
Negative	77 (39.3)	82 (37.3)	0.673			
Positive	119 (60.7)	138 (62.7)				
ES grade, *n* (%)						
I–II	111 (56.6)	89 (40.4)	0.002			
III	79 (40.3)	115 (52.3)		0.16	1.17 (0.73–1.88)	0.506
IV	6 (3.1)	16 (7.3)		0.95	2.59 (0.88–7.64)	0.084
Liver cirrhosis, *n* (%)						
No	93 (47.4)	101 (45.9)	0.753			
Yes	103 (52.6)	119 (54.1)				
No. of nodes, *n* (%)						
1	110 (56.1)	127 (57.7)	0.741			
≥ 2	86 (43.9)	93 (42.3)				
L‐max (cm)						
≤ 5	111 (56.6)	129 (58.6)	0.680			
> 5	85 (43.4)	91 (41.4)				
Tumor margin, *n* (%)						
Smooth	143 (73.0)	84 (38.2)	0.000	1.11	3.04 (1.93–4.80)	0.000
Non‐smooth	53 (27.0)	136 (61.8)				
Tumor growth pattern, *n* (%)						
Intrahepatic growth	116 (59.2)	112 (50.9)	0.091			
Extrahepatic growth	80 (40.8)	109 (49.1)				
Intratumor necrosis, *n* (%)						
Absent	123 (62.8)	130 (59.1)	0.445			
Present	73 (37.2)	90 (40.9)				
Intratumor hemorrhage, *n* (%)						
Absent	180 (91.8)	207 (94.1)	0.367			
Present	16 (8.2)	13 (5.9)				
Pseudo‐capsule, *n* (%)						
Well‐defined capsule	137 (69.9)	77 (35.0)	0.000	1.24	3.47 (2.22–5.42)	0.000
Ill‐defined capsule	59 (30.1)	143 (65.0)				
Peritumoral star node, *n* (%)						
Absent	177 (90.3)	153 (69.5)	0.000	1.07	2.92 (1.59–5.38)	0.001
Present	19 (9.7)	67 (30.5)				

Abbreviations: AFP, alpha‐fetoprotein; ALB, albumin; ALT, alanine aminotransferase; AST, aspartate aminotransferase; CEA, carcinoembryonic antigen; ES grade, Edmondson‐Steiner grade; L‐max, maximum length of tumor; NLR, neutrophil‐to‐lymphocyte ratio; PLR, platelet‐to‐lymphocyte ratio; PLT, platelet count; PT, prothrombin time; RBC, red blood cell count; TBil, total bilirubin.

### 3.2 Feature Selection

**FIGURE 2:**
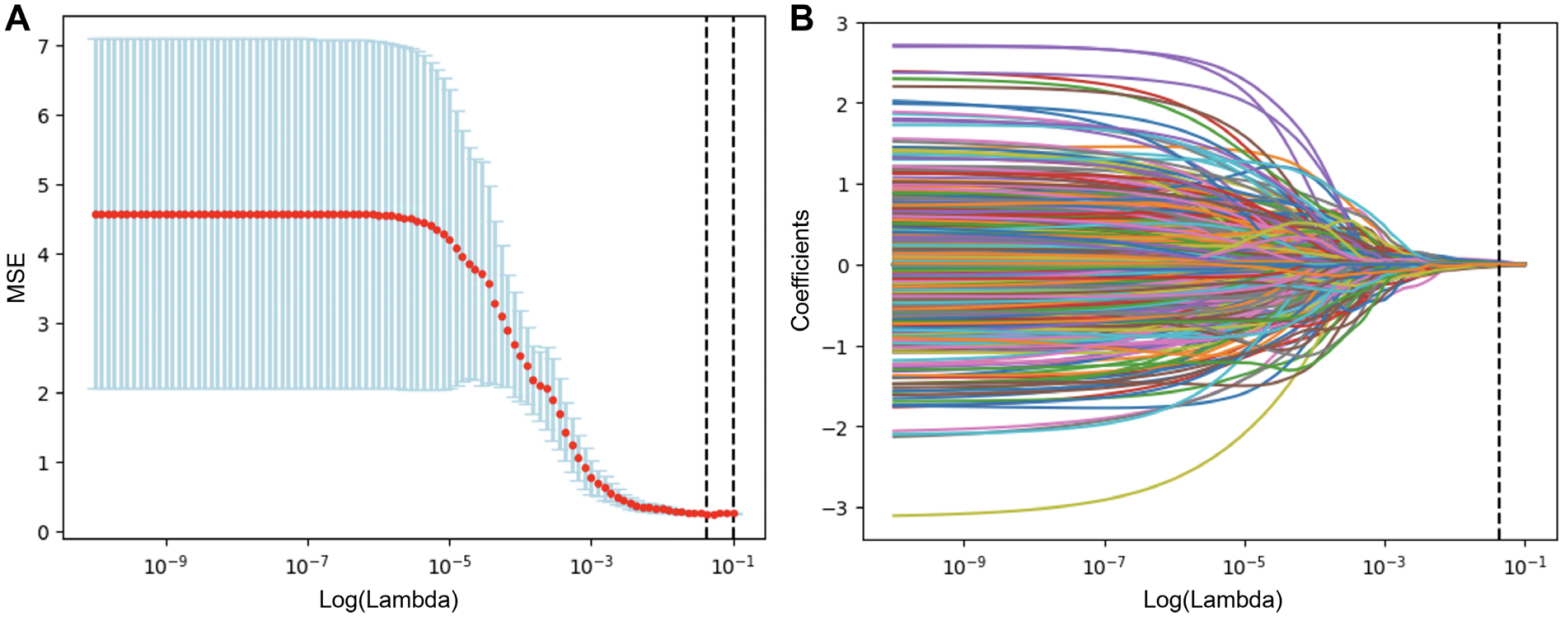
Combined LASSO path analysis. (A) Regularization path showing parameter impact on model performance. (B) Coefficient path depicting feature changes with regularization.

**FIGURE 3:**
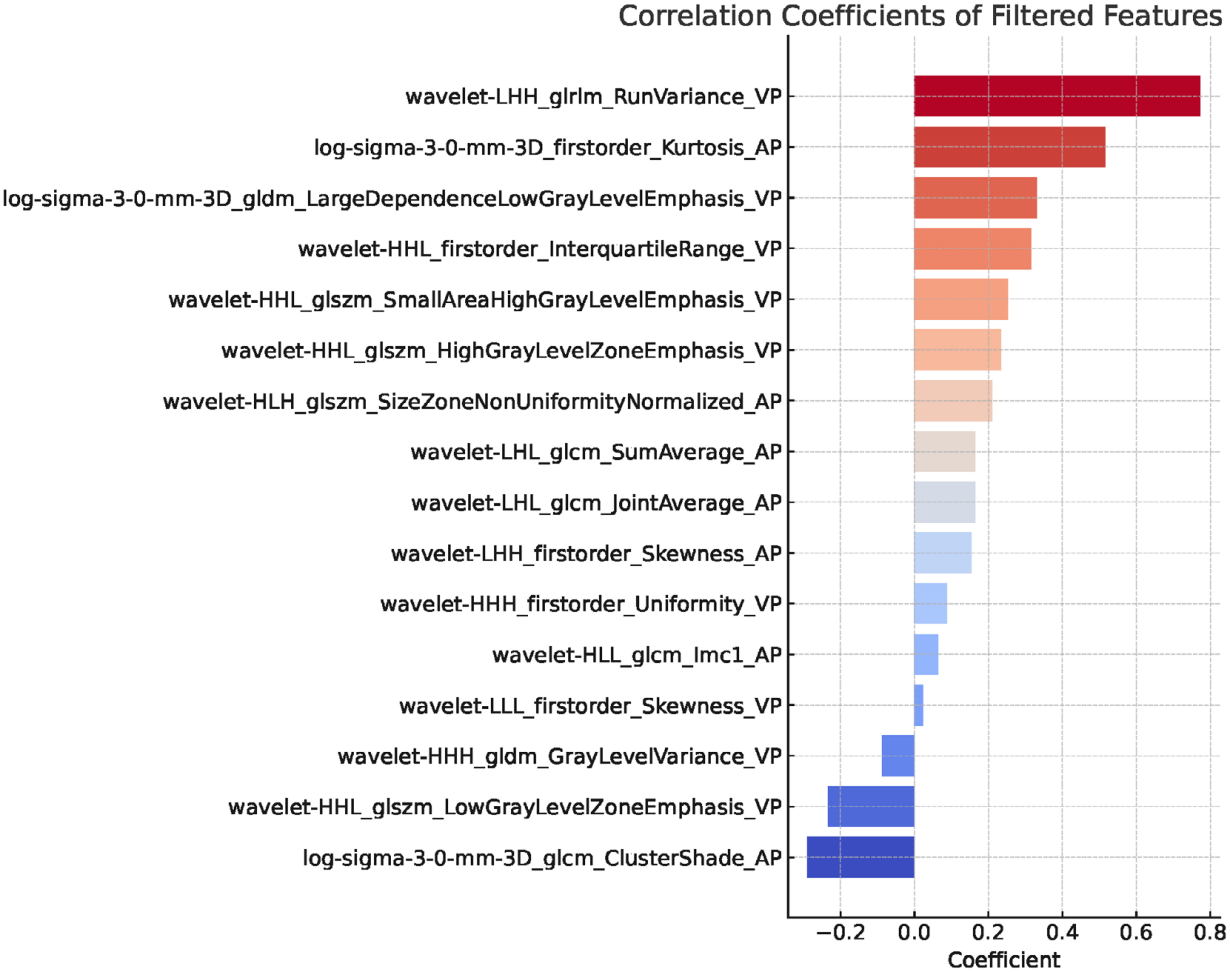
Correlation coefficients of filtered features.

**FIGURE 4:**
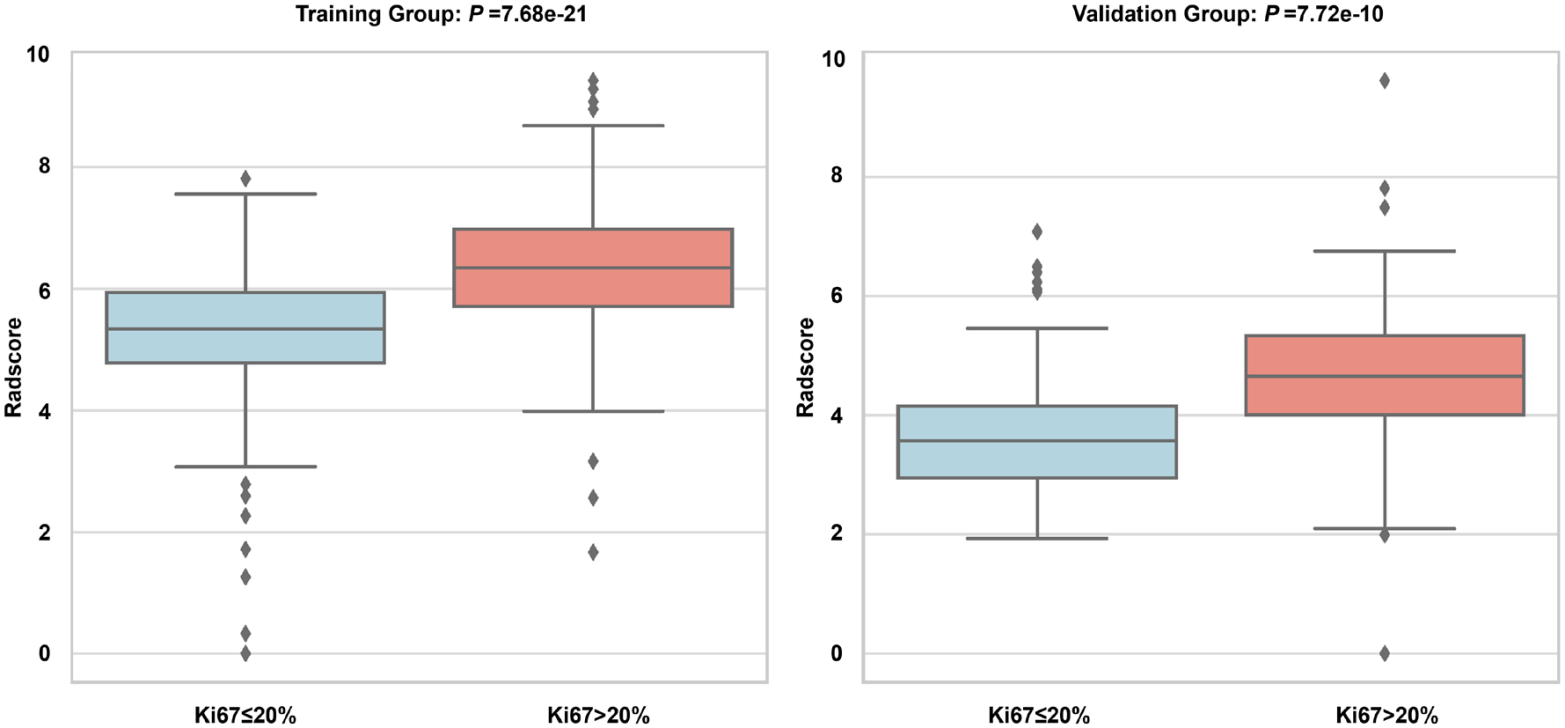
Correlation distribution of Ki‐67 and Radscore.

From the initial extraction of 1441 radiomic features, 1151 features with an ICC value greater than 0.75 were selected post‐validation. Lasso regression was employed for dimension reduction in Figure 2, identifying 16 advanced imaging features, including five first‐order statistical features, four from the GLCM, two from the GLDM, one from the GLRLM, and four from the GLSZM. These features were modeled using a SVM, with coefficients detailed in Table S3 and Figure 3. The calculation formula for Radscore is provided in the Supporting Information—Data S1, and the Radscore distribution across the training and validation groups is shown in Figure 4. Finally, we established a radiomics model based on the Radscore.

### 3.3 Model Performance

**FIGURE 5:**
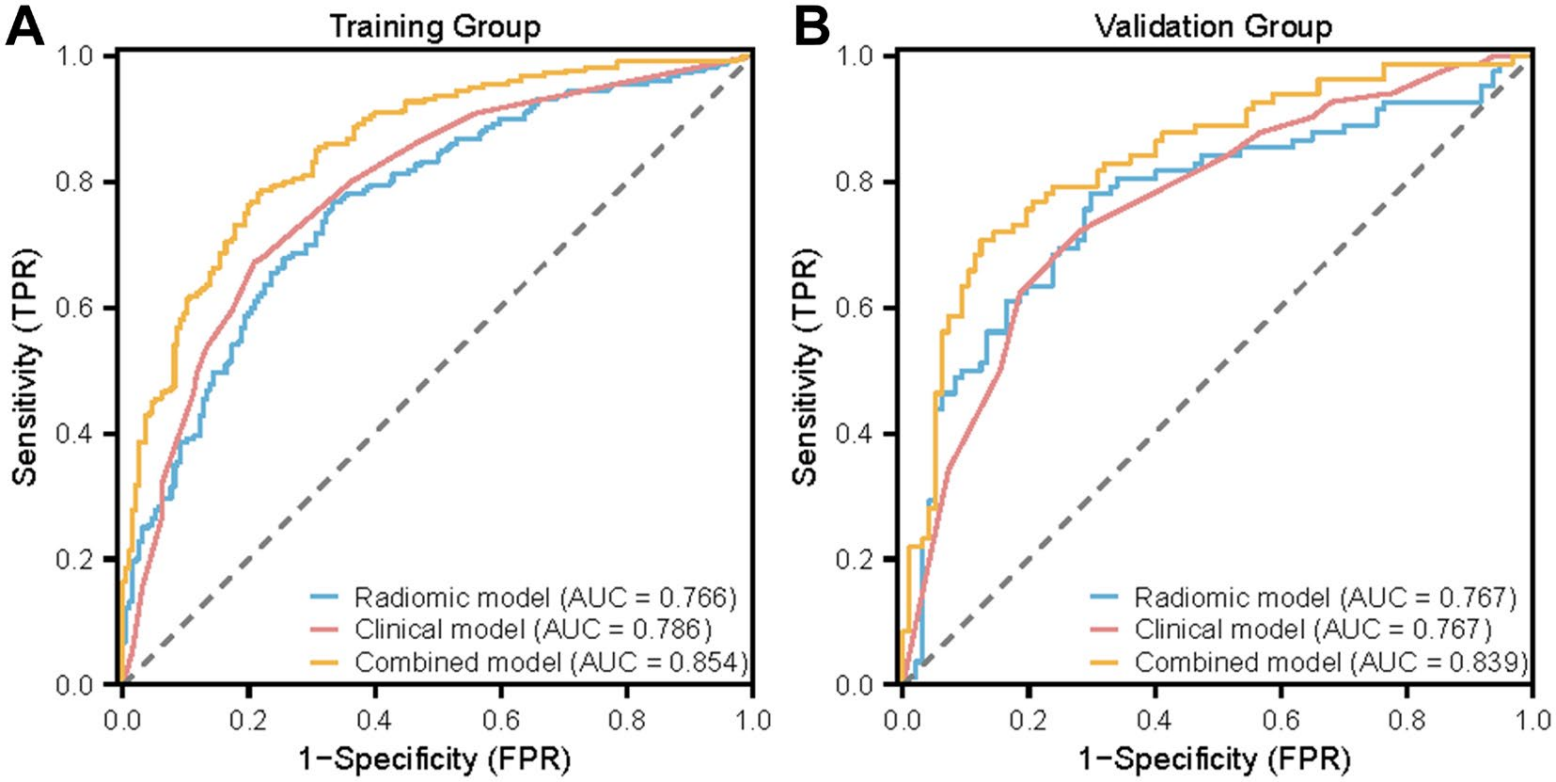
The ROC curves for model Comparison. (A) Clinical model AUC is 0.786, Radiomics model is 0.766, Combined model is 0.854 in the training group. (B) Clinical model AUC is 0.767, Radiomics model is 0.767, combined model is 0.839 in the validation group.

**FIGURE 6:**
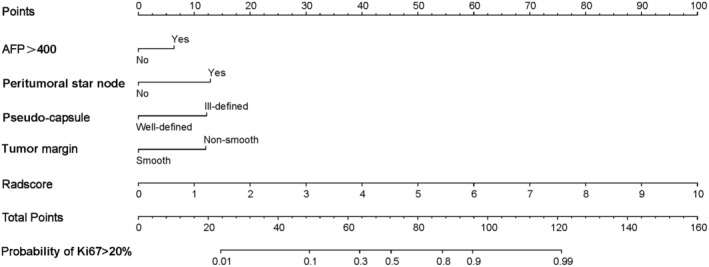
A nomogram for the combined model.

**FIGURE 7:**
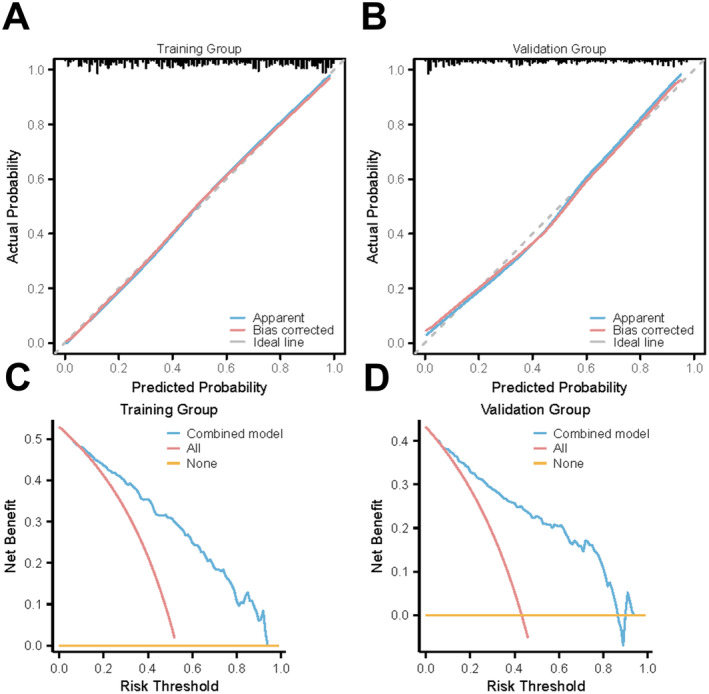
The calibration curves and decision analysis curves. (A) Calibration curve for the training set showing agreement between observed and predicted outcomes. (B) Calibration curve for the validation set illustrating model accuracy. (C) Decision curve analysis for the training set showing clinical net benefit. (D) Decision curve analysis for the validation set demonstrating decision‐making utility.

The clinical model exhibited an AUC of 0.786 in the training group, with a specificity of 67.27% and sensitivity of 79.08%. The validation group's AUC was 0.767, with a specificity of 70.10% and sensitivity of 73.17%. The radiomics model in the training group achieved an AUC of 0.766, a specificity of 73.64%, and a sensitivity of 70.41%, while in the validation group, it recorded an AUC of 0.767, with a specificity of 71.13% and sensitivity of 75.61%. The combined model, incorporating Radscore into the clinical model, achieved an AUC of 0.854 in the training group and 0.839 in the validation group, with specificity and sensitivity rates in Table 2 and Figure 5. A nomogram based on the combined model is presented in Figure 6, with its predictive accuracy evaluated using calibration curves and decision curve analysis in Figure 7.

**TABLE 2:** Comparison of the predictive performance for Ki‐67 of each model.

Models	Training cohort			Validation cohort		
	AUC	Sensitivity	Specitivity	AUC	Sensitivity	Specitivity
Radiomics model	0.770	73.64%	70.41%	0.767	71.13%	75.61%
Clinical model	0.786	67.27%	79.08%	0.740	70.10%	73.17%
Combined model	0.854	78.18%	78.57%	0.833	76.83%	81.44%

### 3.4 Risk Factor Scorecard Model

To facilitate the intuitive use of the established combined model, we assigned scores to Ki‐67‐related risk factors using *β* coefficients from logistic regression. Continuous variables were scored based on quartiles (Q1, Median, Q3) at 4.84, 5.81, and 6.38, respectively in Table 3. The final scores were determined as follows: non‐smooth tumor margin (2 points), ill‐defined capsule (2 points), presence of peritumoral star node (2 points), AFP > 400 (1 point), Radscore [0, 4.84] (2 points), [4.84, 5.81] (3 points), [5.81, 6.38] (5 points), and (6.38, maximum value) (6 points). We calculated the probability *p* = 0.211, representing the maximum Youden index for the model, and determined the threshold score of 9 points after scoring. Consequently, the risk scorecard model indicated that when the score is ≥ 9 points, the patient has a very high risk of high Ki‐67 expression (> 20%) in Table 4.

**TABLE 3:** Multivariate analysis of clinical and radiomic predictors for Ki67 expression in the training group.

Variable	*β*	Wald	OR (95% CI)	*p*
Tumor margin				
Smooth	1.14	20.35	3.12 (1.90–5.11)	0.000
Non‐smooth				
Pseudo‐capsule				
Well‐defined capsule	1.15	21.77	3.16 (1.95–5.13)	0.000
Ill‐defined capsule				
Peritumoral star node				
Absent	1.22	12.86	3.38 (1.74–6.56)	0.000
Present				
AFP (ng/mL)				
≤ 400	0.60	4.74	1.83 (1.06–3.14)	0.030
> 400				
Radscore (4.84, 6.38)	0.94	0.14	2.57 (1.96–3.35)	0.000

**TABLE 4:** Risk factor scorecard model.

Risk factor	Points
Non‐smooth tumor margin	2
Ill‐defined capsule	2
Present peritumoral star node	2
AFP > 400	1
Radscore ≤ 4.84	2
4.84 < Radscore ≤ 5.81	3
5.81 < Radscore ≤ 6.38	5
Radscore > 6.38	6
Threshold score	9

### 3.5 Follow‐Up and Survival

**FIGURE 8:**
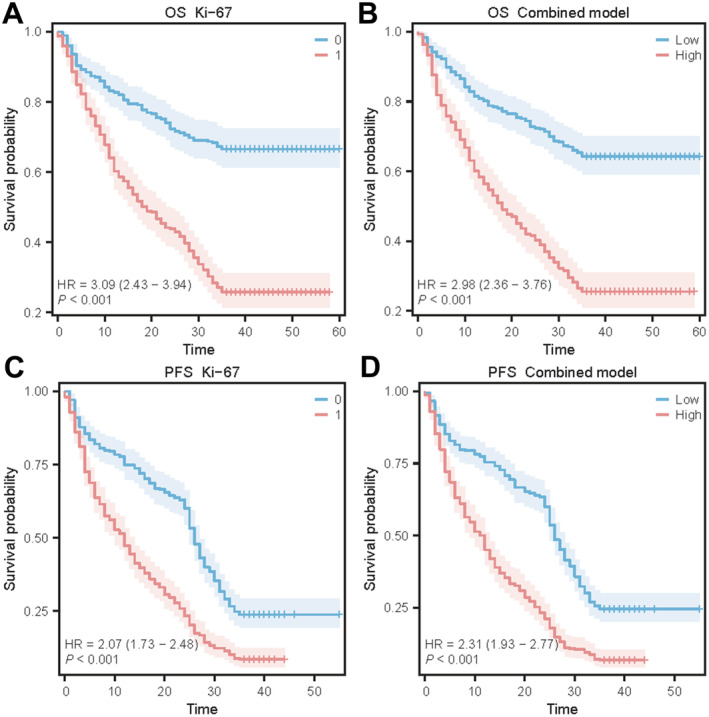
The 3‐year overall survival rates and progression‐free survival rates predictions using histological Ki‐67 and combined model. (A) Kaplan–Meier curves for 3‐year OS for high versus low histological Ki‐67 groups. (B) Kaplan–Meier curves for 3‐year OS for high versus low combined model groups. (C) Kaplan–Meier curves for 3‐year PFS for high versus low histological Ki‐67 groups. (D) Kaplan–Meier curves for 3‐year PFS for high versus low combined model groups.

All patients were followed up for a median duration of 31 months (0–60 months) with a median PFS of 18 months (0–55 months). In the low Ki‐67 group, the 1‐, 2‐, and 3‐year OS rates were 83.09%, 74.10%, and 66.55%, respectively, and the PFS rates were 77.70%, 62.95%, and 23.74%. These rates were significantly higher than in the high Ki‐67 group, which had 1‐, 2‐, and 3‐year OS rates of 64.04%, 44.16%, and 25.87%, and PFS rates of 51.42%, 25.87%, and 8.52%, respectively. Survival data for both groups are in Table S4. Figure 8 shows the Kaplan–Meier curves for 3‐year OS and PFS based on the true Ki‐67 high/low groups and the combined model. Specifically, panels A and C represent the OS and PFS for the true Ki‐67 high and low expression groups, respectively. Panels B and D illustrate the OS and PFS curves based on the predicted Ki‐67 groups using the combined model. These curves demonstrate the model's strong predictive capability.

## 4 Discussion

Ki‐67 has emerged as a crucial biomarker associated with cell proliferation, showing marked expression during the G1, S, G2, and mitotic phases of the cell cycle, while being minimally expressed in the G0 and early G1 phases [21]. This biomarker has demonstrated potential in predicting prognosis and therapeutic responses in various solid tumors, including breast cancer, lung cancer, and HCC. In breast cancer, Ki‐67 expression is utilized to distinguish between luminal A and luminal B subtypes, assess the efficacy of abemaciclib in high‐risk ER‐positive/HER2‐negative breast cancer, and predict systemic chemotherapy responses in neoadjuvant therapy [22]. Research on HCC patients undergoing resection has shown that high Ki‐67 expression correlates with advanced tumor stages and early disease recurrence [7, 23]. Therefore, preoperative prediction of Ki‐67 expression levels is crucial for developing personalized treatment plans, potentially extending patient survival and improving prognosis.

Previous studies have highlighted high Ki‐67 expression as a risk factor for various malignancies [24, 25, 26]. However, a definitive threshold for Ki‐67 remains elusive. Drawing on past research, we used 20% as the cut‐off for Ki‐67 expression and validated this in the survival analysis of HCC patients [14, 19]. Patients with Ki‐67 expression exceeding 20% exhibited markedly lower three‐year overall survival and recurrence‐free survival rates than the low‐expression group. Thus, preoperative non‐invasive assessment of Ki‐67 expression emerges as an imperative task.

In our study, we also found that when Ki‐67 was divided into high and low expression groups, patients in the high Ki‐67 group had significantly worse OS and PFS compared to the low Ki‐67 group after hepatectomy, consistent with previous research. Consequently, we comprehensively analyzed the risk factors for high Ki‐67 expression in HCC patients from clinical aspects, traditional imaging features, and higher‐order imaging characteristics. Through extensive calculations and statistical analyses, we identified ill‐defined pseudo capsules, non‐smooth tumor margins, the presence of peritumoral star nodes, and elevated AFP levels exceeding 400 ng/mL as major risk factors for high Ki‐67 expression. Based on these findings, we established a clinical risk prediction model. Additionally, we conducted radiomics analysis on enhanced CT images, extracting first‐order, second‐order, and higher‐order imaging features. We ultimately selected 16 key features and developed a Radscore formula based on the feature coefficients, transforming image data into numerical data. Using Radscore, we built a radiomics risk prediction model. The combined model, which integrated clinical factors and radiomics features, showed superior performance with an AUC of 0.854 in the training set and 0.839 in the validation set, outperforming the standalone clinical and radiomics models. To better visualize the correlation of risk factors, we converted the combined model into a nomogram. Validation of the nomogram in both the training and validation sets demonstrated perfect calibration and DCA, indicating strong clinical applicability. Furthermore, to facilitate preoperative assessment of high Ki‐67 risk by clinicians, we developed a risk factor scorecard model. This scorecard allows for easy calculation of the probability of high Ki‐67 expression based on specific patient parameters. When the score reaches the threshold, it indicates a high risk of high Ki‐67 expression, potentially necessitating more extensive surgical resection and comprehensive treatment strategies. The scorecard model represents a novel application of radiomics in clinical practice, providing significant positive implications.

Consistent with prior research, we observe that ill‐defined pseudo capsules in imaging studies often indicate invasive or proliferative tendencies in HCC [27]. This aligns with our finding of high Ki‐67 expression in such cases. Similarly, non‐smooth tumor margins, a factor repeatedly linked to poor patient prognosis [28, 29], have been identified as another significant indicator of elevated Ki‐67 expression in our study. The presence of peritumoral star nodes, as previously established, is also a predictor of adverse outcomes in HCC [30]. Moreover, we corroborate the role of AFP levels in anticipating Ki‐67 expression, a marker closely related to reduced PFS and overall survival OS [31, 32]. Additionally, it has been reported in relevant literature that AFP can also predict the therapeutic response to PD‐1 inhibitors [33]. Our findings are in line with existing literature, further substantiating the utility of these factors as prognostic indicators in HCC.

In parallel, in addition to incorporating first and second‐order features, our radiomics model integrates higher‐order features through methods such as wavelet transforms. These advanced features are crucial for accurately depicting the complex internal structure and heterogeneity of HCC tumors, enhancing the model's capacity to detect subtle yet vital tumor characteristics. This approach marks a significant advancement in radiomic analysis, providing a more detailed and nuanced understanding of the tumor's nature. We then utilized the Radscore formula to integrate these features, a method commonly used in other radiomics studies. The median Radscore was 5.81, which effectively quantifies imaging features and enhances the applicability of the radiomics model.

In the study of Ki‐67, Hu et al. employed a deep learning combined radiomics (DLCR) model, collecting data from 108 HCC patients. The inputs included conventional MRI (T2W, DW, and dynamic contrast‐enhanced images) and MRE‐derived shear wave speed (c‐map) and phase angle (φ‐map) images to predict Ki‐67 expression. Their findings indicated that shear wave speed and phase angle are significant predictors, enhancing the model's predictive performance. The c and φ maps from MRE were identified as crucial parameters for assessing tumor proliferation in HCC [12]. Yan et al. developed a random forest (RF) model based on Gd‐EOB‐DTPA‐enhanced MRI radiomics features, predicting high (> 20%) and low (≤ 20%) Ki‐67 expression in 258 HCC patients [19]. Similarly, Qian et al. included 118 HCC patients, extracting radiomics features from intratumoral and peritumoral regions of ultrasound images. Their best model achieved an AUC of 0.870, indicating that integrating intratumoral and peritumoral information enhances the diagnostic performance of the prediction model [15]. Wu et al. constructed a radiomics nomogram using preoperative enhanced CT images from 172 HCC patients (AUC: 0.884 for the training group, 0.819 for the validation group) [14]. Wu H. et al. retrospectively analyzed preoperative CT findings of 74 HCC patients, using MaZda software for texture feature calculations and sequential forward selection for feature selection. Their study employed logistic regression to evaluate the association between texture features and high Ki‐67 (≥ 10%), performing ROC analysis on significant parameters. They found that angular second moment, contrast, correlation, inverse difference moment (IDM), and entropy were highly correlated with Ki‐67 expression. While these studies achieved certain milestones, their applicability and reliability often remain constrained due to limited case numbers. To provide a clearer comparison between our study and existing methods, we have summarized and contrasted key aspects, including radiomics methods, modeling approaches, model performance, and model types from relevant literature. Please refer to Table S5 in the Supporting Information for the detailed comparison. Our study, through a larger sample size analysis, aims to surmount these limitations, offering a more comprehensive perspective for preoperative assessment. Additionally, we not only developed and validated a nomogram model based on the combined model but also established a risk scorecard model. The risk scorecard model aims to provide a simpler and more straightforward method for assessing and calculating the risk level of Ki‐67 expression. This approach is intended to offer insights and guidance for clinical diagnosis and treatment.

While this study contributes to the field of radiomics in HCC, it is not without its limitations. As a single‐center study, the generalizability of our findings might be limited, highlighting the need for multicenter studies to validate and broaden these results. Additionally, the potential gender bias in our cohort, with a higher prevalence of male patients, and the variability in imaging data due to different CT scanner models, are factors that may impact the findings. Despite these considerations, our study's large sample size offers valuable insights, particularly in the preoperative assessment of Ki‐67 expression.

These limitations, however, underscore the necessity for further research. Future studies should aim to address these gaps, ideally through multicenter collaborations involving diverse patient populations and standardized imaging protocols. Such efforts are crucial for advancing the application of radiomics in clinical settings and for the better understanding of its role in HCC management.

## 5 Conclusion

This study highlights the effectiveness of combining radiomics features with clinical factors to predict Ki‐67 expression in HCC. The integrated model, which includes imaging features from enhanced CT and clinical risk factors, showed superior performance in predicting Ki‐67 expression compared to standalone models. The development of a risk scorecard model provides a practical tool for clinicians to assess preoperative Ki‐67 expression risk, aiding in personalized treatment planning. Despite the study's limitations, including its single‐center design and potential gender bias, the findings offer valuable insights into the use of radiomics in HCC management. Future multicenter studies are needed to validate these results and standardize imaging protocols, enhancing the clinical applicability of radiomics in HCC.

## Author Contributions

**Chiyu Cai:** conceptualization‐equal, data curation‐equal, formal analysis‐equal, investigation‐equal, methodology‐equal, software‐lead, writing – original draft‐lead; **Liancai Wang:** project administration‐equal, resources‐equal, resources‐equal, writing – review and editing‐equal, writing – review and editing‐equal; **Lianyuan Tao:** resources‐equal, software‐equal, validation‐equal; **Hengli Zhu:** investigation‐equal, supervision‐equal; **Yongnian Ren:** formal analysis‐equal, software‐equal; **Deyu Li:** conceptualization‐lead, funding acquisition‐equal, project administration‐equal, resources‐equal, writing – review and editing‐equal; **Dongxiao Li:** conceptualization‐equal, methodology‐equal, supervision‐equal.

## Ethics Statement

This research strictly complied with the ethical standards and principles outlined in the Declaration of Helsinki. It received approval from the Ethics Committee of Zhengzhou University People's Hospital, documented under the reference ((2023) Ethic Review No. (12)). The nature of this study was retrospective, involving the analysis of existing patient data extracted from medical records. Given its retrospective nature and the use of pre‐existing data, the requirement for obtaining informed consent was formally waived by the aforementioned Ethics Committee, as noted in the same approval document.

## Conflicts of Interest

The authors declare no conflicts of interest.

## Supporting information


Data S1.

## Data Availability

The data that support the findings of this study are not publicly available due to ethical restrictions, but are available from the corresponding author upon reasonable request.
