# Supplementary material for: Imaging‐Based Prediction of Ki‐67 Expression in Hepatocellular Carcinoma: A Retrospective Study
Source: Cancer Med. 2025 Feb 18;14(4):e70562. doi: 10.1002/cam4.70562 (PMC11834164; doi:10.1002/cam4.70562)
Supplement: Supplementary file 1 — Data S1. [file CAM4-14-e70562-s001.docx]

**Supplementary material**

1. **CT Scanning Protocol**

In the conducted research, each participant was subjected to a comprehensive abdominal multiphasic dynamic contrast-enhanced CT scan. The scanning process utilized a selection of MDCT (multi-detector computed tomography) units, including GE Healthcare’s LightSpeed VCT and Discovery CT750 HD, Siemens' SOMATOM Definition Flash, and Philips' Brilliance 16. The imaging protocol incorporated two distinct phases: the arterial (AP) and the venous phase (VP). For contrast, Ultravist 370 (produced by Bayer Schering Pharma, Berlin, Germany) was administered intravenously, dosed at 1.5 mL per kilogram of the patient's body weight. The administration rate was maintained between 3.0 to 3.5 mL/s, facilitated by the Ulrich CT Plus 150 syringe pump (Ulrich Medical, Ulm, Germany). Timing for the AP scan was set within the window of 25 to 30 seconds subsequent to the injection of the contrast medium, whereas the VP scan was scheduled at an interval of 60 to 70 seconds post-injection. The scanning parameters were meticulously calibrated, featuring a 120 kV setting, automated control of tube current, and variable rotation times (0.4, 0.5, or 0.75 seconds). Detector configurations were varied, including 64×0.625 mm, 128×0.625 mm, and 16×0.625 mm options, coupled with a field of view stretching from 300 to 500 mm in both dimensions. The imaging matrix was configured at 512×512, and the slices were consistently maintained at 5 mm intervals with an equal thickness, whereas the thickness for reconstructed sections was precisely set at 2 mm.

1. **CT Image Quality Evaluation Criteria**

| **Item** | **Evaluation content and method** | **Deduction points** |
| --- | --- | --- |
| Image contrast | Compare the CT image to a reference image; poor contrast quality | 5 |
| Image layering | Assess the clarity of the layers in the CT image | 5 |
| Scanning coverage | Excessive or incomplete coverage | 5 |
| Artifacts from  objects | Such as artifacts caused by unremoved metallic items | 10 |
| Motion artifacts | No impact on diagnosis | 5-10 |
| Equipment artifacts | No impact on diagnosis | 5-10 |
| Contrast enhancement effect | Suboptimal but does not impact diagnosis | 10-15 |
| Image marking | Incomplete labeling | 5 |
| Errors in key labels | Such as incorrect left/right labeling, name, or gender | 50 |
| Positional requirement | CT images should be taken with specific positioning | 5 |
| Photograph order | Not following the standard sequence | 5 |
| Exam location | Inconsistency between the examination order and the actual examination location | 50 |

**Image Quality Scoring Methodology:** Based on CT imaging quality standards, each CT image is scored out of 100 points. Deductions are made based on the above criteria. Scoring levels: Excellent ≥ 90 points, Good 80-89 points, Fair 70-79 points, and Unsatisfactory < 70 points. < 70 points is considered poor image quality for the CT scan.

1. **Formula**

**Formula of Z-score Normalization**

*Z=σ(X−μ)​*

*X* is the original pixel value, *μ* is the average of all pixel values in the image, *σ* is the standard deviation.

**Formula of Trilinear Interpolation**

*V′(x,y,z)=∑_i,j,k_V(i,j,k)×w_ijk_​*

*V(i,j,k)* is the value of neighboring voxels, *w_ijk_* is a weight based on distance.

**Formula of interclass correlation coefficient (ICC)**

*ICC(2,1)=​MS_B_−MS_W_ / MS_B_+(k−1)×MS_W_​​*

*MS_B_* is the average sum of squares between groups,*MS_W_* is the sum of squares within the group,*K* is the number of evaluators.

First, we constructed a multivariate logistic regression model by incorporating major risk factors into the regression model to estimate the regression coefficients (β values) for each risk factor.

**Linear Combination of the Logistic Regression Model**

*Z=β_0_+∑^n^ _i=1_ β_i_X_i_*

*Z* is the linear combination of the logistic regression model, *β_0_* is the intercept, *β_i_* is the regression coefficient for each risk factor, and *X_i_* is the corresponding risk factor.

**Points Calculation for Each Group**

*Points_ij_=(W_ij_−W_REF_)*β_i_)/B*

*W_ij_* is the reference value for each group, *W_REF_* is the baseline risk reference value,

*β_i_* is the regression coefficient, and *B* is a constant (0.6).

**Youden Index Calculation**

*J=Sensitivity+Specificity−1*

*P=1/1+e^−z^​*

*z=ln(1−P)/P​*

The threshold with the highest Youden index was selected as the optimal threshold.

J is the Youden Index, P is the predicted probability corresponding to the optimal threshold.

1. **Calculation formula of Radscore**

*Radscore=∑(Feature Value×Coefficient)×Slope+Intercept*

Radscore=(0.516×log-sigma-3-0-mm-3D_firstorder_Kurtosis_AP-0.289×log-sigma-3-0-mm-3D_glcm_ClusterShade_AP+0.330×log-sigma-3-0-mm-3D_gldm_LargeDependenceLowGrayLevelEmphasis_VP+0.164×wavelet-LHL_glcm_JointAverage_AP+0.164×wavelet-LHL_glcm_SumAverage_AP+0.154×wavelet-LHH_firstorder_Skewness_AP)+0.773×wavelet-LHH_glrlm_RunVariance_VP+0.064×wavelet-HLL_glcm_Imc1_AP+0.210×wavelet-HLH_glszm_SizeZoneNonUniformityNormalized_AP+0.316×wavelet-HHL_firstorder_InterquartileRange_VP+0.253×wavelet-HHL_glszm_SmallAreaHighGrayLevelEmphasis_VP+0.234×wavelet-HHL_glszm_HighGrayLevelZoneEmphasis_VP-0.234×wavelet-HHL_glszm_LowGrayLevelZoneEmphasis_VP+0.087×wavelet-HHH_firstorder_Uniformity_VP-0.087×wavelet-HHH_gldm_GrayLevelVariance_VP+0.023×wavelet-LLL_firstorder_Skewness_VP)×0.2284+5.8336

**Table S1**  Patients clinical characteristics

| **Variable** |  | **Training cohort(N=416)** | |  | **Validation cohort(N=179)** | |  | P value | |
| --- | --- | --- | --- | --- | --- | --- | --- | --- | --- |
| **Sex,n(%)** |  |  |  |  |  |  |  |  |  |
| Male |  | 335(80.5) | |  | 150(83.8) | |  | 0.346 | |
| Female |  | 81(19.5) | |  | 29(16.2) | |  |  |  |
| **Age(y)** |  |  |  |  |  |  |  |  |  |
| ≤65 |  | 306(73.6) | |  | 138(78.4) | |  | 0.363 | |
| ＞65 |  | 110(26.4) | |  | 41(21.6) | |  |  |  |
| **ALT(U/L)** |  |  |  |  |  |  |  |  |  |
| ≤40 |  | 266(63.9) | |  | 102(57.0) | |  | 0.109 | |
| ＞40 |  | 150(36.1) | |  | 77(43.0) | |  |  |  |
| **AST(U/L)** |  |  |  |  |  |  |  |  |  |
| ≤40 |  | 247(59.4) | |  | 93(52.0) | |  | 0.093 | |
| ＞40 |  | 169(40.6) | |  | 86(48.0) | |  |  |  |
| **ALB(g/L)** |  |  |  |  |  |  |  |  |  |
| ≤40 |  | 224(53.8) | |  | 110(61.5) | |  | 0.086 | |
| ＞40 |  | 192(46.2) | |  | 69(38.5) | |  |  |  |
| **TBil(umol/L)** |  |  |  |  |  |  |  |  |  |
| ≤17.1 |  | 271(65.1) | |  | 125(69.8) | |  | 0.266 | |
| ＞17.1 |  | 145(34.9) | |  | 54(30.2) | |  |  |  |
| **NLR,n(%)** |  |  |  |  |  |  |  |  |  |
| ≤2.5 |  | 200(48.1) | |  | 89(49.7) | |  | 0.713 | |
| ＞2.5 |  | 216(51.9) | |  | 90(50.3) | |  |  |  |
| **PLR,n(%)** |  |  |  |  |  |  |  |  |  |
| ≤111.5 |  | 212(51.0) | |  | 91(50.8) | |  | 0.978 | |
| ＞111.5 |  | 204(49.0) | |  | 88(49.2) | |  |  |  |
| **RBC(×10^12^/L)** |  |  |  |  |  |  |  |  |  |
| ≤3 |  | 12(2.9) | |  | 3(1.7) | |  | 0.388 | |
| ＞3 |  | 404(97.1) | |  | 176(98.3) | |  |  |  |
| **PLT(×10^9^/L)** |  |  |  |  |  |  |  |  |  |
| ≤100 |  | 100(24.0) | |  | 44(24.6) | |  | 0.887 | |
| ＞100 |  | 316(76.0) | |  | 135(75.4) | |  |  |  |
| **PT(s)** |  |  |  |  |  |  |  |  |  |
| ≤13 |  | 260(62.5) | |  | 113(63.1) | |  | 0.884 | |
| ＞13 |  | 156(37.5) | |  | 66(36.9) | |  |  |  |
| **CEA(ng/mL)** |  |  |  |  |  |  |  |  |  |
| ≤5 |  | 371(89.2) | |  | 154(86.0) | |  | 0.274 | |
| ＞5 |  | 45(10.8) | |  | 25(14.0) | |  |  |  |
| **AFP(ng/mL)** |  |  |  |  |  |  |  |  |  |
| ≤400 |  | 294(70.7) | |  | 120(67.0) | |  | 0.377 | |
| ＞400 |  | 122(29.3) | |  | 59(33.0) | |  |  |  |
| **HBsAg,n(%)** |  |  |  |  |  |  |  |  |  |
| Negative |  | 159(38.2) | |  | 71(39.7) | |  | 0.740 | |
| Positive |  | 257(61.8) | |  | 108(60.3) | |  |  |  |
| **ES grade,n(%)** |  |  |  |  |  |  |  |  |  |
| Ⅰ-Ⅱ |  | 200(48.1) | |  | 73(40.8) | |  | 0.146 | |
| Ⅲ |  | 194(46.6) | |  | 99(55.3) | |  |  |  |
| Ⅳ |  | 22(5.3) | |  | 7(3.9) | |  |  |  |
| **Liver cirrhosis,n(%)** |  |  |  |  |  |  |  |  |  |
| No |  | 194(46.6) | |  | 87(48.6) | |  | 0.659 | |
| Yes |  | 222(53.4) | |  | 92(51.4) | |  |  |  |
| **Ki-67(%)** |  |  | |  |  |  |  |  |  |
| ≤20 |  | 196(47.1) | |  | 82(45.8) | |  | 0.770 | |
| ＞20 |  | 220(52.9) | |  | 97(54.2) | |  |  |  |

**Abbreviation:** ALT: alanine aminotransferase; AST: aspartate aminotransferase; ALB: albumin; TBil: total bilirubin; NLR: neutrophil-to-lymphocyte ratio; PLR: platelet-to-lymphocyte ratio; RBC: red blood cell count; PLT: platelet count; PT: prothrombin time; CEA: carcinoembryonic antigen; AFP: alpha-fetoprotein

ES grade: Edmondson-Steiner grade

**Table S2** Patients imaging characteristics

| **Variable** |  | **Training cohort** | |  | **Validation cohort** | |  | P **value** | |
| --- | --- | --- | --- | --- | --- | --- | --- | --- | --- |
| **No. of nodes,n(%)** |  |  |  |  |  |  |  |  |  |
| 1 |  | 237(57.0) | |  | 108(60.3) | |  | 0.446 | |
| ≥2 |  | 179(43.0) | |  | 71(39.7) | |  |  |  |
| **L-max(cm)** |  |  |  |  |  |  |  |  |  |
| ≤5 |  | 240(57.7) | |  | 93(52.0) | |  | 0.196 | |
| ＞5 |  | 176(42.3) | |  | 86(48.0) | |  |  |  |
| **Tumor margin,n(%)** |  |  |  |  |  |  |  |  |  |
| smoooth |  | 227(54.6) | |  | 93(52.0) | |  | 0.784 | |
| non-smooth |  | 189(45.4) | |  | 86(48.0) | |  |  |  |
| **Tumor growth pattern,n(%)** |  |  |  |  |  |  |  |  |  |
| intrahepatic growth |  | 228(54.8) | |  | 110(61.5) | |  | 0.133 | |
| extrahepatic growth |  | 188(45.2) | |  | 69(39.5) | |  |  |  |
| **Intratumor necrosis,n(%)** |  |  |  |  |  |  |  |  |  |
| absent |  | 253(60.8) | |  | `109(60.9) | |  | 0.986 | |
| present |  | 163(39.2) | |  | 70(39.1) | |  |  |  |
| **Intratumor hemorrhage,n(%)** |  |  |  |  |  |  |  |  |  |
| absent |  | 387(93.0) | |  | 167(93.3) | |  | 0.906 | |
| present |  | 29(7.0) | |  | 12(6.7) | |  |  |  |
| **Pseudo-capsule,n(%)** |  |  |  |  |  |  |  |  |  |
| well-defined capsule |  | 214(51.4) | |  | 91(50.8) | |  | 0.892 | |
| ill-defined capsule |  | 202(48.6) | |  | 88(49.2) | |  |  |  |
| **Peritumoral star node,n(%)** |  |  |  |  |  |  |  |  |  |
| absent |  | 330(79.3) | |  | 145(81.0) | |  | 0.640 | |
| present |  | 86(20.7) | |  | 34(19.0) | |  |  |  |

**Abbreviation:** L-max: maximum length of tumor.

**Table S3** Filtered feature

| **Filtered features** |  |  |  | **Coefficient** |
| --- | --- | --- | --- | --- |
| log-sigma-3-0-mm-3D_firstorder_Kurtosis_AP |  |  |  | 0.516 |
| log-sigma-3-0-mm-3D_glcm_ClusterShade_AP |  |  |  | -0.289 |
| log-sigma-3-0-mm-3D_gldm_LargeDependenceLowGrayLevelEmphasis_VP |  |  |  | 0.330 |
| wavelet-LHL_glcm_JointAverage_AP |  |  |  | 0.164 |
| wavelet-LHL_glcm_SumAverage_AP |  |  |  | 0.164 |
| wavelet-LHH_firstorder_Skewness_AP |  |  |  | 0.154 |
| wavelet-LHH_glrlm_RunVariance_VP |  |  |  | 0.773 |
| wavelet-HLL_glcm_Imc1_AP |  |  |  | 0.064 |
| wavelet-HLH_glszm_SizeZoneNonUniformityNormalized_AP |  |  |  | 0.210 |
| wavelet-HHL_firstorder_InterquartileRange_VP |  |  |  | 0.316 |
| wavelet-HHL_glszm_SmallAreaHighGrayLevelEmphasis_VP |  |  |  | 0.253 |
| wavelet-HHL_glszm_HighGrayLevelZoneEmphasis_VP |  |  |  | 0.234 |
| wavelet-HHL_glszm_LowGrayLevelZoneEmphasis_VP |  |  |  | -0.234 |
| wavelet-HHH_firstorder_Uniformity_VP |  |  |  | 0.087 |
| wavelet-HHH_gldm_GrayLevelVariance_VP |  |  |  | -0.087 |
| wavelet-LLL_firstorder_Skewness_VP |  |  |  | 0.023 |

**Table S4** The 3-year overall survival rate and progress free survival rate of the training group and the validation group

|  |  |  | **1-year** | **2-year** | **3-year** |
| --- | --- | --- | --- | --- | --- |
| **Training set** | Ki67≤20% | PFS | 70.73% | 56.10% | 20.73% |
|  |  | OS | 76.83% | 68.29% | 59.76% |
|  | Ki67>20% | PFS | 53.61% | 28.87% | 13.40% |
|  |  | OS | 68.04% | 44.33% | 30.93% |
| **Validation set** | Ki67≤20% | PFS | 80.61% | 65.82% | 25.00% |
|  |  | OS | 85.71% | 76.53% | 69.39% |
|  | Ki67>20% | PFS | 50.45% | 24.55% | 6.36% |
|  |  | OS | 62.27% | 44.09% | 23.63% |

**Table S5** Comparison of Radiomics-Based Predictive Models for Ki-67 Expression in Hepatocellular Carcinoma

| **Researchers** | **Sample size** | **Type of imaging used** | **Feature selection and modeling method** | **Model performance** | **Key predictive clinical features** | **Model type** |
| --- | --- | --- | --- | --- | --- | --- |
| Ye Z. et al. ^[1]^ (2019) | 89 | MRI | LASSO+LR | Radiomics model: AUC: 0.878 Clinical model: AUC: 0.795 Combined model: AUC: 0.936 | AFP, BCLC-stage, Capsule integrity, Tumor margin, Enhancing capsule | Nomogram |
| Hu X. et al.^[2]^  (2022) | 108 | MRI | LASSO+SVM | Radiomics model: AUC: 0.8 Clinical model: AUC: NA Combined model: AUC: 0.9 | AFP | LR model |
| Yan Y. et al.^[3]^  (2024) | 258 | MRI | RFE+RF | Radiomics model: AUC: 0.872; Accuarcy:0.728; Precision: 0.735; Recall: 0.722 Clinical model: AUC: 0.780; Accuarcy:0.694; Precision: 0.694; Recall: 0.693 Combined model: AUC: 0.876; Accuarcy:0.739; Precision: 0.747; Recall: 0.733 | AFP, Tumor size, Growth type, Peritumoral enhancement | LR model |
| Wu C. et al.^[4]^ (2022) | 172 | CT | ANOVA+Mann-Whitney U test+correlation analysis+GBDT | Radiomics model: AUC: 0.854; Sensitivity: 0.873; Specificity: 0.684 Clinical model:NA Combined model: AUC: 0.884; Sensitivity: 0.778; Specificity: 0.877 | AFP, Edmondson grade | Nomogram |
| Qian H. et al.^[5]^ (2023) | 118 | Ultrasound | T-test+LASSO+SVM/RF/KNN/LR/ANN | Intratumoral model: AUC: 0.796; Sensitivity: 0.947; Specificity: 0.588; Accuarcy:0.780; Precision: 0.720  Peritumoral model: AUC: 0.772; Sensitivity: 0.421; Specificity: 1.000; Accuarcy:0.694; Precision: 1.000 Clinical model: NA Combined model1: AUC: 0.87; Sensitivity: 0.737; Specificity: 0.882; Accuarcy:0.806; Precision: 0.875  Combined model2: AUC: 0.762; Sensitivity: 0.790; Specificity: 0.647; Accuarcy:0.722; Precision: 0.714 | Age | LR model |
| Wu H. et al.^[6]^ (2020) | 74 | CT | SFS+LR | Contrast model: AUC: 0.823; Sensitivity: 0.889; Specificity: 0.750 Correlation model: AUC: 0.777; Sensitivity: 0.852; Specificity: 0.800 Combined model: AUC: 0.836; Sensitivity: 0.963; Specificity: 0.750 | NA | LR model |
| Dong Y. et al.^[7]^ (2022) | 101 | Enhanced Ultrasound | MRMR+LASSO+LR | BMUS model: AUC: 0.805; Sensitivity: 0.933; Specificity: 0.545 S-CEUS model: AUC: 0.908; Sensitivity: 0.933; Specificity: 0.818 | NA | LR model |
| Fan Y. et al.^[8]^ (2021) | 151 | MRI | LASSO+LR | Radiomics model: AUC: 0.880; Sensitivity: 0.862; Specificity: 0.826 Clinical model:NA Combined model: AUC: 0.922; Sensitivity: 0.987; Specificity: 0.783 | AFP | LR model |
| Zhao Y. et al.^[9]^ (2023) | 208 | CT | LASSO+LR | Radiomics model: AUC: 0.762; Sensitivity: 0.870; Specificity: 0.670 Clinical model: AUC: 0.836; Sensitivity: 0.860; Specificity: 0.790 Combined model: AUC: 0.903; Sensitivity: 0.987; Specificity: 0.783 | AFP, Non-rim arterial phase hyperenhancement, Portal vein tumor thrombus, Two-trait predictor of venous invasion | LR model |
| Zhang D. et al.^[10]^ (2024) | 310 | Ultrasound | LASSO+LR | Radiomics model: AUC: 0.752; Sensitivity: 0.700; Specificity: 0.750 Clinical model: AUC: 0.797; Sensitivity: 0.495; Specificity: 0.976 Combined model: AUC: 0.870; Sensitivity: 0.731; Specificity: 0.857 | AFP, Tumor differentiation | Nomogram |
| Zhang L. et al.^[11]^ (2023) | 244 | Ultrasound | RFE+XGBoost+SVM | Radiomics model: AUC: 0.955; Sensitivity: 0.973; Specificity: 0.810 Clinical model: AUC: 0.809; Sensitivity: 0.685; Specificity: 0.685 Combined model: AUC: 0.986; Sensitivity: 0.973; Specificity: 0.869 | NA | LR model |
| **Our study** | **595** | **CT** | **LASSO+SVM+LR** | **Radiomics model: AUC: 0.770; Sensitivity: 0.736; Specificity: 0.704 Clinical model: AUC: 0.786; Sensitivity: 0.673; Specificity: 0.791 Combined model: AUC: 0.854; Sensitivity: 0.782; Specificity: 0.786** | **AFP, Tumor margin, Pseudo-capsule, Peritumoral star node** | **Nomogram+Risk factor scorecard** |

**Abbreviation:** AFP: Alpha-Fetoprotein; ANN: Artificial Neural Network; ANOVA: Analysis of Variance; AUC: Area Under the Curve; BCLC-stage: Barcelona Clinic Liver Cancer Stage; BMUS: B-mode Ultrasound; GBDT: Gradient Boosting Decision Tree; KNN: K-Nearest Neighbors; LASSO: Least Absolute Shrinkage and Selection Operator; LR: Logistic Regression; MRMR: Minimum Redundancy Maximum Relevance; RF: Random Forest; RFE: Recursive Feature Elimination; S-CEUS: Contrast-Enhanced Ultrasound; SFS: Sequential Forward Selection; SVM: Support Vector Machine


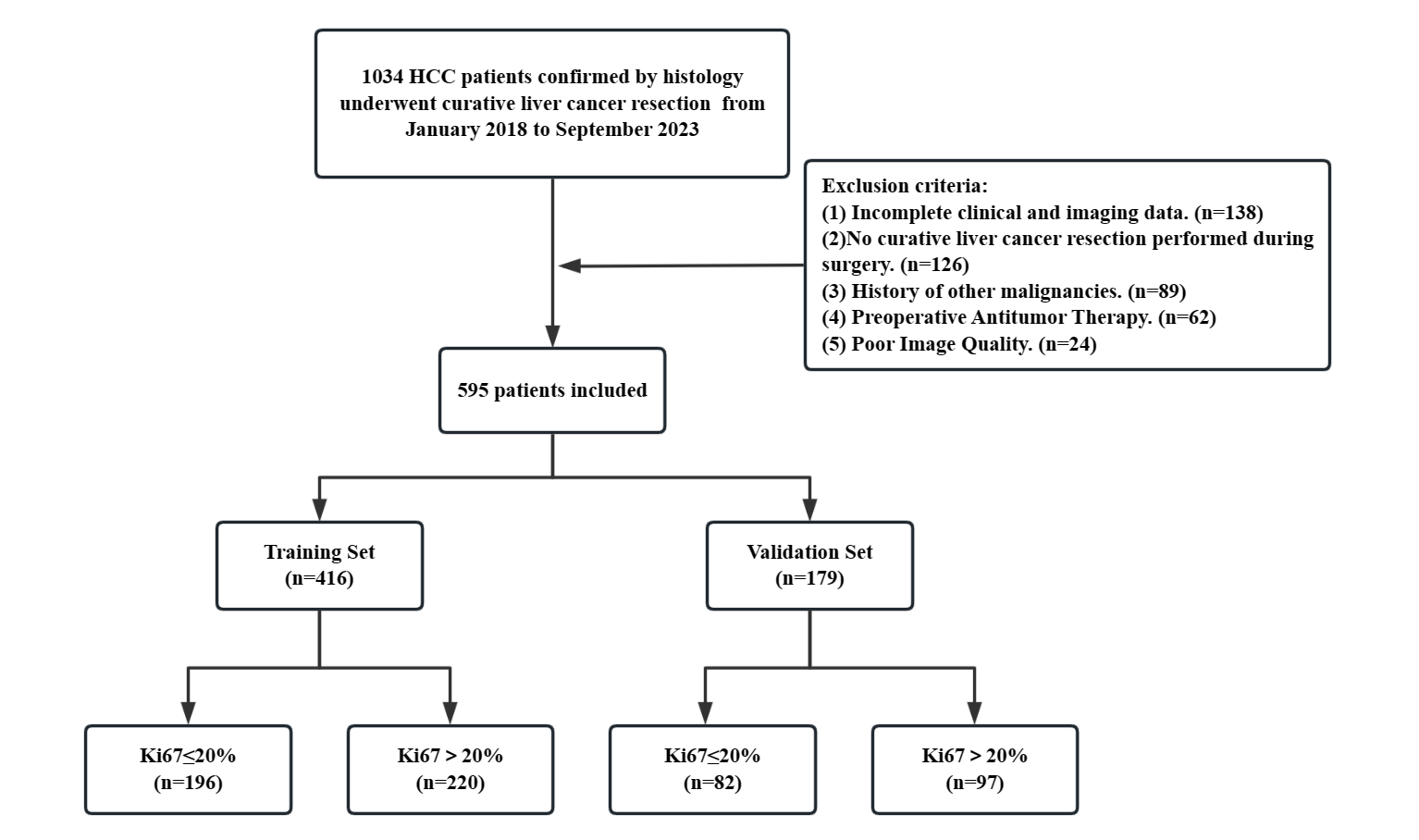


**Fig S1.** Participant screening flowchart.


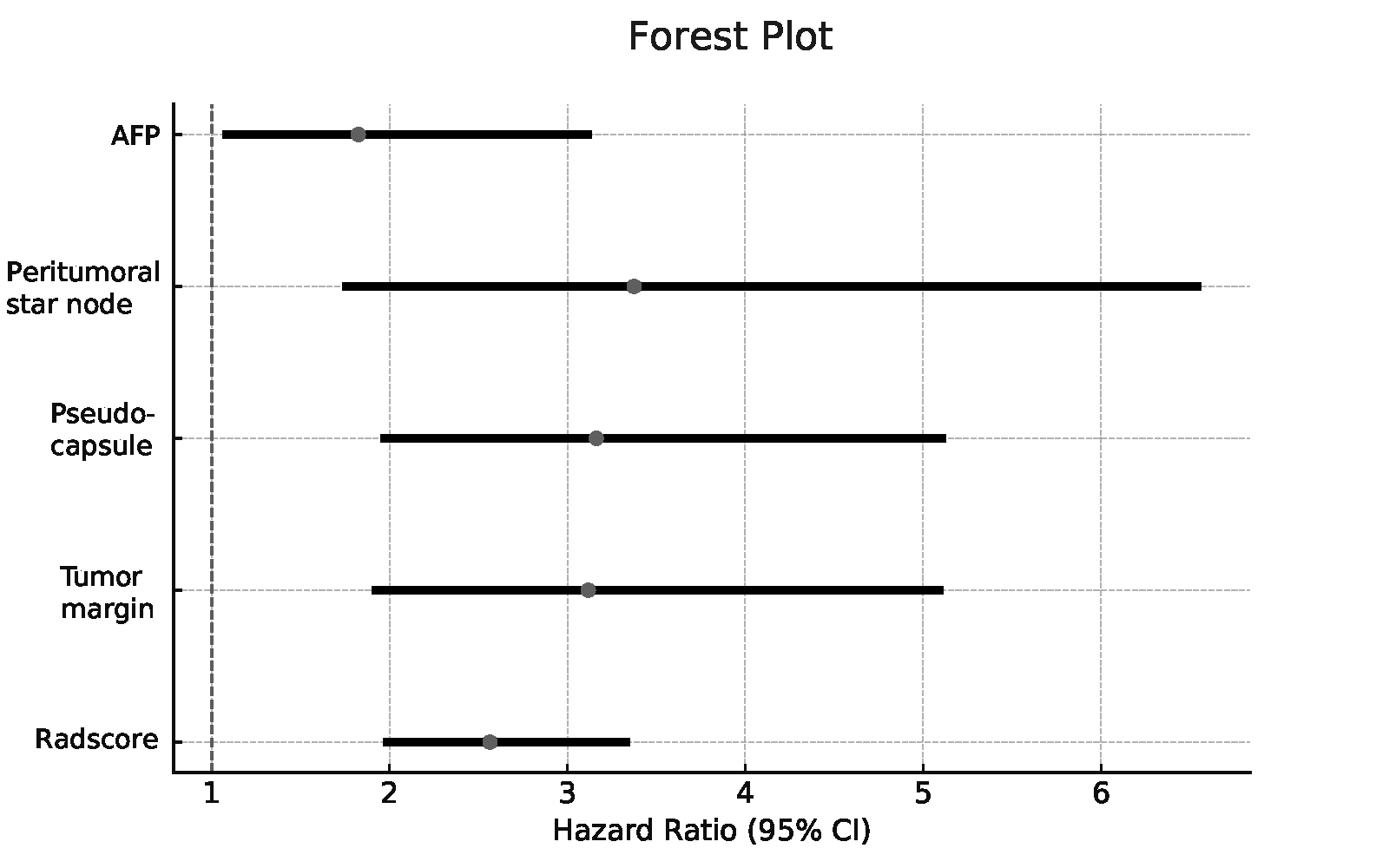


**Figure S2.** Forest plot of multivariable analysis results

**Reference**

[1] YE Z, JIANG H, CHEN J, et al. Texture analysis on gadoxetic acid enhanced-MRI for predicting Ki-67 status in hepatocellular carcinoma: A prospective study [J]. Chin J Cancer Res, 2019, 31(5): 806-17.

[2] HU X, ZHOU J, LI Y, et al. Added Value of Viscoelasticity for MRI-Based Prediction of Ki-67 Expression of Hepatocellular Carcinoma Using a Deep Learning Combined Radiomics (DLCR) Model [J]. Cancers (Basel), 2022, 14(11).

[3] YAN Y, LIN X S, MING W Z, et al. Radiomic Analysis Based on Gd-EOB-DTPA Enhanced MRI for the Preoperative Prediction of Ki-67 Expression in Hepatocellular Carcinoma [J]. Acad Radiol, 2024, 31(3): 859-69.

[4] WU C, CHEN J, FAN Y, et al. Nomogram Based on CT Radiomics Features Combined With Clinical Factors to Predict Ki-67 Expression in Hepatocellular Carcinoma [J]. Front Oncol, 2022, 12: 943942.

[5] QIAN H, SHEN Z, ZHOU D, et al. Intratumoral and peritumoral radiomics model based on abdominal ultrasound for predicting Ki-67 expression in patients with hepatocellular cancer [J]. Front Oncol, 2023, 13: 1209111.

[6] WU H, HAN X, WANG Z, et al. Prediction of the Ki-67 marker index in hepatocellular carcinoma based on CT radiomics features [J]. Phys Med Biol, 2020, 65(23): 235048.

[7] DONG Y, ZUO D, QIU Y J, et al. Prediction of Histological Grades and Ki-67 Expression of Hepatocellular Carcinoma Based on Sonazoid Contrast Enhanced Ultrasound Radiomics Signatures [J]. Diagnostics (Basel), 2022, 12(9).

[8] FAN Y, YU Y, WANG X, et al. Radiomic analysis of Gd-EOB-DTPA-enhanced MRI predicts Ki-67 expression in hepatocellular carcinoma [J]. BMC Med Imaging, 2021, 21(1): 100.

[9] ZHAO Y M, XIE S S, WANG J, et al. Added value of CE-CT radiomics to predict high Ki-67 expression in hepatocellular carcinoma [J]. BMC Med Imaging, 2023, 23(1): 138.

[10] ZHANG D, ZHANG X Y, LU W W, et al. Predicting Ki-67 expression in hepatocellular carcinoma: nomogram based on clinical factors and contrast-enhanced ultrasound radiomics signatures [J]. Abdom Radiol (NY), 2024, 49(5): 1419-31.

[11] ZHANG L, DUAN S, QI Q, et al. Noninvasive Prediction of Ki-67 Expression in Hepatocellular Carcinoma Using Machine Learning-Based Ultrasomics: A Multicenter Study [J]. J Ultrasound Med, 2023, 42(5): 1113-22.
